# Evaluation of autophagic and apoptotic markers during infection with animal virus causing hemorrhagic fever in rabbits

**DOI:** 10.3389/fmicb.2024.1517725

**Published:** 2025-01-08

**Authors:** Dominika Bębnowska, Sylwia Rzeszotek, Agnieszka Kolasa, Karolina Wiśniewska, Magdalena Żabińska, Aneta Szulc, Zuzanna Cyske, Karolina Pierzynowska, Aleksandra Wilk, Paulina Niedźwiedzka-Rystwej

**Affiliations:** ^1^Institute of Biology, University of Szczecin, Szczecin, Poland; ^2^Center for Experimental Immunology and Immunobiology in Infectious Disease and Cancer, University of Szczecin, Szczecin, Poland; ^3^Department of Histology and Embryology, Faculty of Medicine and Dentistry, Pomeranian Medical University in Szczecin, Szczecin, Poland; ^4^Department of Molecular Biology, Faculty of Biology, University of Gdansk, Gdansk, Poland

**Keywords:** *Lagovirus* europaeus, rabbit haemorrhagic disease, autophagy, apoptosis, rabbits

## Abstract

**Introduction:**

*Lagovirus europaeus*/GI.1 and GI.2 cause severe Rabbit Haemorrhagic Disease, and immune processes are among the important pathomechanisms of the disease. Autophagy and apoptosis are two key mechanisms involved in the host antiviral response. Both of these processes have been characterized in infection with GI.1 strains, while data on infection with GI.2 strains still need to be studied. This is particularly important because infection with different strains is associated with a different host immune response profile.

**Methods:**

In this work, we analyzed the expression of selected genes and proteins involved in autophagic flux in the liver, spleen, kidney and peripheral blood, but also apoptotic cell death in the liver and peripheral blood of rabbits infected with the GI.2 strain.

**Results:**

As a result, we showed that autophagy is strongly activated in the liver, spleen and kidney of infected rabbits, and confirmed the activation of apoptosis in the liver.

**Discussion:**

This study highlights the role of apoptosis and autophagy in the immune response in rabbits infected with Lagovirus europaeus/GI.2.

## Introduction

Autophagy and apoptosis are processes responsible for maintaining homeostasis in organisms in response to stressful stimuli. Autophagy can be described as a mechanism of cell survival, as the role of this process amounts to the degradation and recycling of cytoplasmic components, but also of intracellular pathogens (Parzych and Klionsky, 2014). Viral infection and the associated viral replication are an important stress factor for the cell. For this reason, autophagy is a process often activated in infectious states as an antiviral mechanism. In addition to removing invading viruses, at later stages of infection autophagy promotes the processing of viral antigens by facilitating the induction of adaptive immune responses (Choi et al., 2018). Apoptosis is the best-studied mechanism of programmed death that is responsible for the removal of unwanted cells. Viral infection is one of the stimuli that triggers apoptotic death in the cell, which in general limits replication and release of progeny viruses. However, excessive or abnormal apoptosis often leads to the development of disease and is therefore, in many cases, part of the essential mechanisms of viral pathogenesis (Thomson, 2001). The action of many stress stimuli leads to the induction of both processes in the cell. There is ample evidence that autophagy and apoptotic machinery work together, antagonize or support each other, so that together they determine the fate of the cell (Mariño et al., 2014). The current state of knowledge makes it clear that both of these processes are crucial in the context of viral infections. For this reason, understanding the relationship between autophagic and apoptotic responses during infection is an important line of research that contributes to understanding the role of these processes in the course and pathogenesis of disease.

Rabbit Haemorrhagic Disease (RHD) is a disease caused by infection with *Lagovirus europaeus*/GI.1 and GI.2 and was first described over 30 years ago (Liu et al., 1984). In 2010, *Lagovirus europaus*/GI.2 emerged and was classified as the second genotype of rabbit haemorrhagic fever virus (Bębnowska and Niedźwiedzka-Rystwej, 2019; Le Gall-Reculé et al., 2011). The specific features of this disease, such as hepatocyte damage resulting from viral replication, cause RHD to be successfully used as an animal model of acute liver failure (ALF). In addition, it has been described that this disease shares several similarities with viral haemorrhagic fever (VHF) in other species and can therefore serve as a research model for VHF (Bębnowska and Niedźwiedzka-Rystwej, 2022). Although the disease has been well characterized and it is known that during RHD, disseminated intravascular coagulation (DIC) occurs, as well as apoptosis and necrosis in many organs, the exact mechanism of pathogenesis is still incompletely understood (Abrantes et al., 2012). However, studies have shown that an important feature of the infection is also an alteration of the host immune potential, largely due to apoptosis of lymphocytes (Niedźwiedzka-Rystwej and Deptuła, 2023; Niedźwiedzka-Rystwej and Deptuła, 2012), granulocytes (Niedźwiedzka-Rystwej et al., 2013), and monocytes (Alonso et al., 1998). The occurrence of autophagy in RHD has been confirmed in the liver in infection with GI.1 strain (Vallejo et al., 2014). Given that the relationship between infection and autophagy and apoptosis may take different shapes, further studies are important to fully understand the relationship between autophagy and apoptosis in *Lagovirus europaeus* infection. For this reason, this study aimed to evaluate the activity of autophagic and apoptotic markers in key organs and blood samples of rabbits infected with *Lagovirus europaeus*/GI.2.

## Materials and methods

### Experimental infection

The animal experiment was carried out at the Pomeranian Medical University in Szczecin under the approval of the Local Ethical Committee for Animal Experiments in Poznań No. 35/2022.

Non- vaccinated against *Lagovirus europaeus*/GI.1 and GI.2 European rabbits *Oryctolagus cuniculus*/Crl:KBL (NZW)/0052 were used in the experiment. The animals were purchased from a licensed breeder (AnimaLab Sp. z o.o., Poznań, Poland). Twenty animals (6 months old) with body weights in the range of 4.5 kg (±10%) were used in the study. The animals were randomly divided into two groups: a control group (*n* = 10; 50% males and 50% females) and an experimental group infected with the *Lagovirus europaeus*/GI.2 strain (*n* = 10; 50% males and 50% females). After delivery to the laboratory, the animals underwent adaptation (3-week period). In their cages, they had access to hay balls and wooden blocks as environmental enrichment. The animals were maintained under standard laboratory conditions.

The virus strain used in the study was isolated from a rabbit that died because of naturally acquired infection in Italy (RHDV2_Ri2017 strain; GenBank: OQ680671). The inoculum (1 mL) intramuscular administered to animals from the experimental group was prepared according to the method described by us earlier (Niedźwiedzka-Rystwej and Deptuła, 2023). Infection was provoked by the intramuscular injection of 2 × 10^4^ haemagglutination units of the virus (Vallejo et al., 2014; Tunon et al., 2003) [assuming that 1 HA unit corresponds to 10^4^ particles per mL (Ryu, 2017)], which was determined by the haemagglutination assay as described earlier (Bębnowska et al., 2023). The hemagglutination titer was obtained at a 1:16 dilution. The animals from the control group received the same amount of placebo (1 mL) in the form of PBS (phosphate-buffered saline).

The experiment started with the administration of the inoculum, and this moment was marked as 0 h of the experiment. The health status of all rabbits, including rectal measurement of body temperature, was monitored at least twice a day from the time of inoculate injection. During the experiment blood was collected from each animal into an anticoagulant (heparin) tube (1 mL) at selected timepoints: 0, 12, 24, 36 and 48 h post inoculation (h p.i.). The onset of severe symptoms of the disease was considered the terminal moment of the experiment. Animals qualified for euthanasia were anesthetized by intravenous injection of sodium pentobarbital at a dose of 90 mg/kg, and then cardiac arrest was induced by administration of sodium pentobarbital at a dose of 250 mg/kg. Tissue samples (from liver, spleen, and kidney) were collected from rabbits immediately after death (both from animals that died suddenly during the experiment and those euthanized) and were preserved in RNAlater stabilizing solution (Thermo Fisher Scientific, Waltham, Massachusetts, United States) and then stored at −80°C until analysis.

### Real-time PCR

Immediately after blood collection at selected timepoints erythrocytes in peripheral blood were lysed using Red Blood Cell Lysis Buffer (A&A Biotechnology, Gdańsk, Poland), using the protocol provided by the producer. Total RNA was isolated from PBL pellet and tissue samples using RNeasy Mini Kit (A&A Biotechnology, Poland) according to the manufacturer’s instructions. The quantity and quality of the isolate were measured using NanoDrop ™ 2000 spectrophotometer (Thermo Fisher Scientific, Waltham, Massachusetts, United States). To obtain cDNA the RevertAid First Strand cDNA Synthesis Kit (Thermo Fisher Scientific, Waltham, Massachusetts, United States) was used as recommended by the manufacturer.

To analyze relative gene expression quantitative real-time PCR was performed using the LightCycler® 480 Instrument II (Roche Diagnostics GmbH, Mannheim, Germany) and the PowerUp™ SYBR™ Green Master Mix Thermo Fisher Scientific, Waltham, Massachusetts, United States according to manufacturer’s recommendations. In the study, specific primers were used (Table 1). Obtained threshold cycle (Ct) values were normalized by *β-actin* gene expression, and the relative gene expression was calculated using the Pfaffl method (Pfaffl, 2001). To determine viral genome copies a dilution series of full-length *L. europaeus* GI.2 transcript standards was prepared (ranging from 1 × 10^6^ copies/μl to 1 × 10^1^ copies/μl). A standard curve and absolute quantification were performed using a LightCycler 480 II instrument.

**Table 1:** Primers used in the study (Vallejo et al., 2014; Sudo and Minami, 2011; Chen et al., 2019; Duarte et al., 2015).

Gen	Sense primer (5′-3 ′)	Antisense primer (5′-3 ′)
*Beclin-1*	CATGCAATGGTGGCTTTCC	TCTCGCCCTTTTCAACCTCTT
*UVRAG*	GCGGCGTCTTCGACATCT	GATGGCCGTTTCTATTAACAATGTT
*Atg5*	CGTCCTGTGGCTGCAGATG	AAGGACACACTTCTTTGAGGAGATC
*Atg12*	TGCTGAAGGCTGTGGGAGAT	TGTTCGCTCTACAGCCCATTT
*Atg16L1*	CCACCAAACCGGCATGAG	CTTGCAGCTGGCTGTCATTC
*MAP1LC3B*	AAGACCTTCAAGCAGCGCC	CACGTGGTCGGGTACAAGGA
*Caspase-3*	GCTGGACAGTGGCATCGAGA	TCCGAATTTCGCCAGGAATAGTAA
*Bcl-2*	GATTGTGGCCTTCTTTGAGTTC	AAGTCTTCAGAGACACCCAGGA
*Bax*	GTGTCTCAAGCGCATTGGCG	CAAACATGTCGGCCTGCCACT
*β-actin*	TGGCATCCTGACGCTCAA	TCGTCCCAGTTGGTCACGAT
RHDV2	TGGAACTTGGCTTGAGTGTTGA-3	ACAAGCGTGCTTGTGGACGG

### Immunohistological methods

The dissected intestinal tissues were fixed with 10% formalin for at least 24 h, but for no more than 36 h, and then treated as described previously (Bębnowska et al., 2024). For IHC reactions we used ImmPRESS HRP Universal (Horse Anti-Mouse/Rabbit IgG), PLUS Polymer Kit, Peroxidase (Vector Laboratories, Newark, CA, United States; cat. no. 7800). To expose the epitopes to IHC procedure, deparaffinized and rehydrated sections were boiled in Target Retrieval Solution (DakoCytomation, Glostrup, Denmark, cat. no. S2369) in a water bath (99°C for 8 min.). Once cooled and washed with PBS, the endogenous peroxidase activity was blocked using peroxidase blocking reagent. Antibodies were diluted in Antibody Diluent with Background Reducing Components (Dako, Dako, Santa Clara, CA, United States, S302 S3022). Slides were incubated for 1 h at room temperature (RT), with primary antibodies against LC3b (anti-LC3b- antibody, rabbit polyclonal, cat# NB100-2220, Novus Biologicals, Centennial, CO, United States); against Atg12 (anti-Atg12 antibody, rabbit polyclonal, cat# orb153327, Biorbyt, Cambridge, United Kingdom) and against Beclin-1 (anti-Beclin 1 antibody, cat# orb625103, rabbit polyclonal, Biorbyt, Cambridge, United Kingdom). Antibodies were diluted in Antibody Diluent (cat# ab64211, Abcam, Cambridge, United Kingdom). To visualize the antigen–antibody complex, sets of reagent HRP-related system were used, based on the reaction of avidin-biotin-horseradish peroxidase with DAB as a chromogen, according to included staining procedure instructions. Sections were washed in distilled H_2_O and counterstained with hematoxylin (Mar-Four, Konstantynów Łódzki, Poland). For a negative control, specimens were processed in the absence of primary antibodies. Positive staining was determined microscopically (Leica DM5000B, Wetzlar, Germany) by visual identification of brown pigmentation. The experiments were repeated independently twice.

### Quantitative computer image analysis histological slides

Micrographs of tissues (liver, kidney, and spleen) stained with antibodies against LC3b, Atg12, and Beclin-1 were analyzed with the use of ImageJ Fiji software.^1^ First, the downloaded image was opened in Fiji software and color deconvolution was performed (Image>Color>Color Deconvolution), and the hematoxyline + DAB option was selected. Next, the threshold was selected (Image>Adjust>Treshold); the minimum threshold value was set at zero and the maximum threshold value was adjusted so that the background signal was removed, without removing the true signal from the hematoxyline + DAB-stained tissues. The percentage of area covered by the pigmentation was measured (Analyze > Measure). Three independent analyses (with a slightly changed maximum threshold) were performed and the results were averaged. The area of tissue where preparation led to bending of the specimen or tissue damage was not included in the analysis.

### Western blot analysis

Levels of selected apoptotic proteins were estimated from tissue sample extracts treated with T-PER™ Tissue Protein Extraction Reagent (Cat. No. 78510, ThermoScientific™) and a mixture of protease and phosphatase inhibitors (Roche Applied Science, Penzberg, Germany; #05892791001 and #11873580001), and Bead Ruptor Elite (Cat. No. SKU 19-040E, Omni international). The samples were centrifuged for 10 min (12,000 rpm, 4°C). Protein lysates were transferred to new Eppendorf tubes. Analysis of protein levels in lysates was conducted using a Bradford reagent. Proteins were separated using the WES system (WES - Automated Western Blots with Simple Western; ProteinSimple, San Jose, California, United States), with a 12–230 kDa separation module (#SM-W003), and detected using an Anti-Mouse (#DM-002) or Anti-Rabbit (#DM-001) detection module, according to the manufacturer’s instructions. The Total protein module (#DM-TP01, ProteinSimple, San Jose, CA, United States) was used as a loading control. The following primary antibodies were used in the study: cleaved caspase-3 (Asp175) (5A1E) rabbit mAb (#9664, Cell Signaling Technology), cleaved caspase-6 (Asp162), rabbit mAb (#9761, Cell Signaling Technology), and cleaved PARP (Asp214) (D64E10) Rabbit mAb (no. 5625, Cell Signaling Technology), LAMP1 (#9091, Cell Signaling), LAMP2 (#49067, Cell Signaling), Beclin-1 (#3495S, Cell Signaling), p62/SQSTM1 (sc-48402, Santa Cruz Biotechnology) 1:50 and LC3 LC3A/LC3B (sc-398822, Santa Cruz Biotechnology) 1:250.

### Caspase-3 activity

Caspase-3 Activity Assay Kit (#5723, Cell Signaling Technology) was used to determine caspase-3 activity according to the recommendation of the manufacturer. Samples with a protein content of 0.5 mg were prepared for the experiment. EnSpire (PerkinElmer, Waltham, Massachusetts, United States) device was used for fluorescence measurement using excitation at 380 nm and emission at 440 nm wavelength and with a gain value of 60.

### Statistical analysis

To perform statistical analysis Tibco Statistica 13.3 (StatSoft, Palo Alto, CA, USA) was used. The values of the parameters were presented as arithmetic means, standard deviations (SD), and standard error (SE). The Shapiro–Wilk test was used to test the normal distribution of continuous variables. Student’s t-test was used to compare data of 2 groups data with a normal distribution and the Mann–Whitney U test was used for data with a non-normal distribution.

## Results

### Clinical signs of rabbit haemorrhagic disease and post-mortem analysis

Clinical signs observation showed that some animals died suddenly at 12–36 h p.i. without any symptoms of disease (*n* = 6). From 24 h p.i. remaining animals (*n* = 4) showed apathy, conjunctival congestion, dysponoea, fever >41°, anorexia, and the human endpoints of the experiment reached 36–48 h p.i. Post-mortem analysis confirmed the characteristic signs of RHD in all animals.

### Viral genome copies

**Figure 1:**
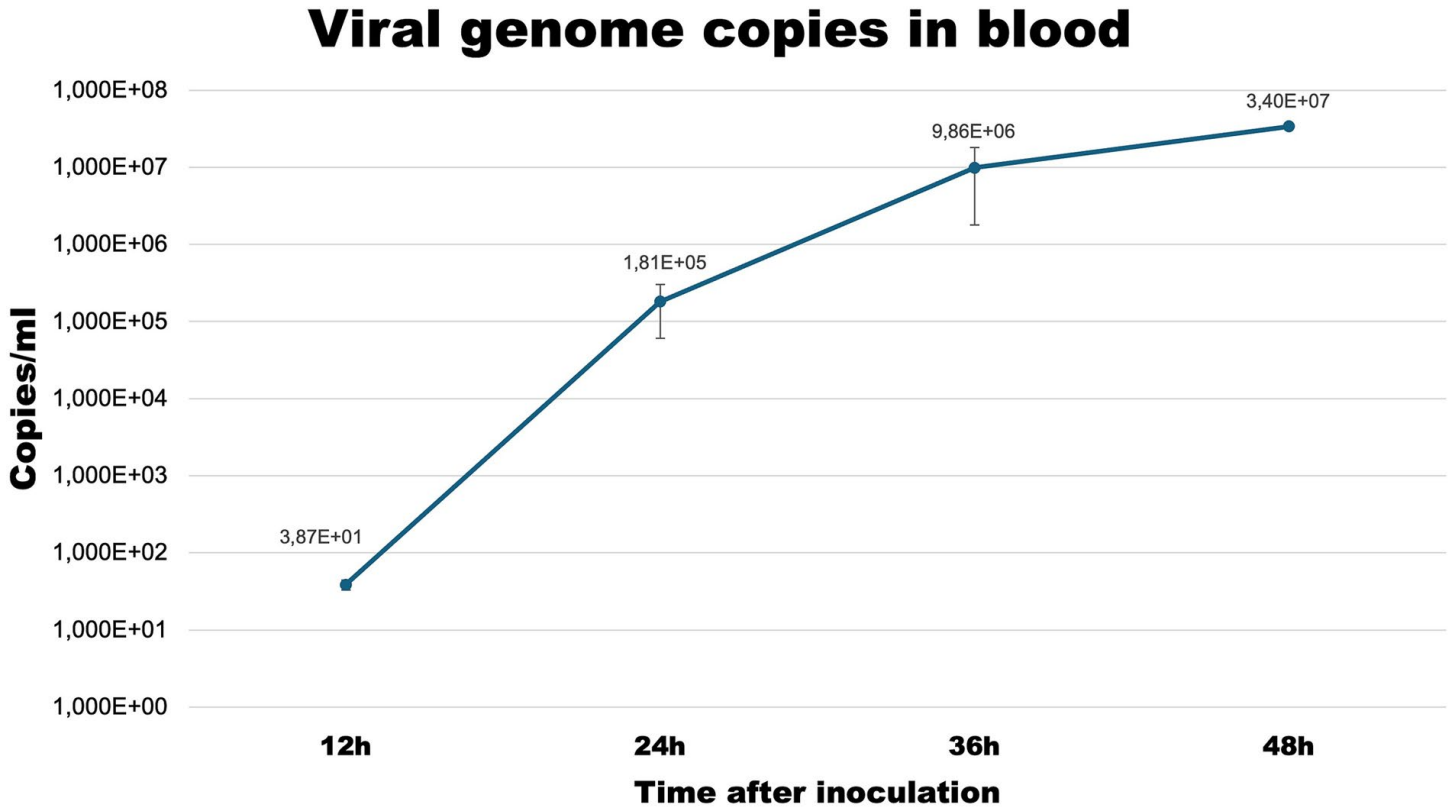
Viral copies/ml of blood samples collected at selected timepoints from infected rabbits.

To confirm the effectiveness of the infection protocol we determined the level of viral load in blood samples at each timepoint (Figure 1).

### Activation of apoptosis occurs during *Lagovirus europaeus*/GI.2 infection

**Figure 2:**
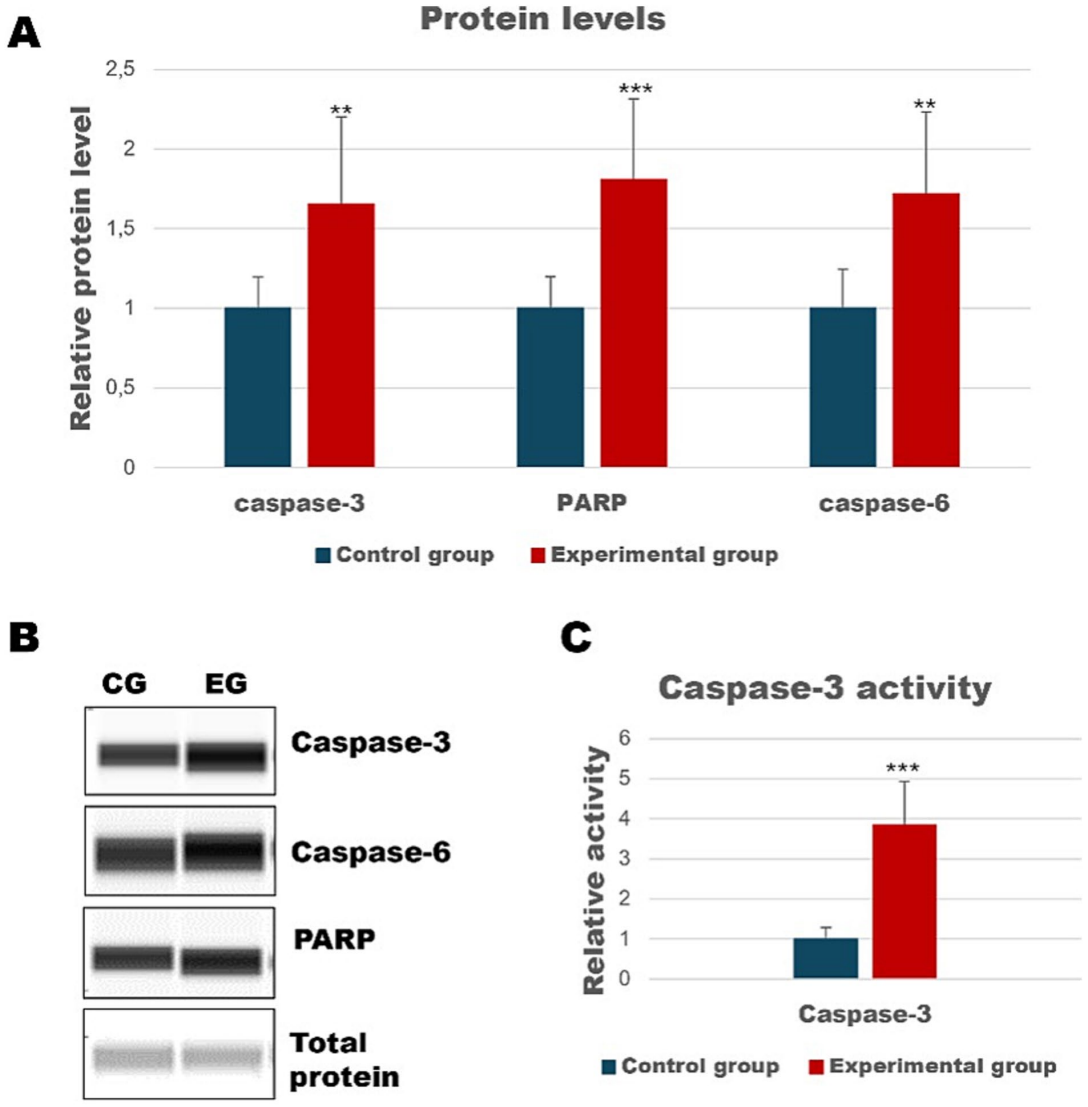
Relative levels of cleaved proteins in liver samples measured by the Western Blot method **(A)**. Panel **(B)** showed representative blots of cleaved apoptotic proteins. Panel **(C)** shows the activity of caspase-3 determined by colorimetric assay. All values are expressed as mean value ± SD. ^**^p ≤ 0.03; ^***^p ≤ 0.01. CG-control group; EG-experimental group.

Insights into apoptotic death in selected organs of rabbits infected with *Lagovirus europaeus*/GI.2 have been the subject of our previous studies (Bębnowska et al., 2023; Bębnowska et al., 2024). However, given that the liver is a central organ targeted by the virus, we decided to further explore the characteristics of this process. In this study, we demonstrated an increased amount of cleaved caspase-3 and caspase-6 (executioner caspases) in the experimental group compared to the control (*p* ≤ 0.03; Figure 2). In addition, we also determined the level of PARP protein, whose cleavage leads to activation of the apoptotic pathway via the intrinsic pathway (Chaitanya et al., 2010). Our results showed that there was an increased amount of cleaved PARP in the liver of infected rabbits (*p* ≤ 0.01; Figure 2). Since caspase-3 is a key performer of apoptosis, we also decided to determine its activity. As a result, our results indicated that caspase-3 activity in the liver is increased in infected rabbits compared to control (*p* ≤ 0.01; Figure 2).

Executor caspase-3 is one of the main effectors of apoptosis, which is involved in both apoptosis induced by the extrinsic death receptor-mediated pathway and the mitochondrial pathway (Thomson, 2001). The Bax and Bcl-2 proteins belong to the regulatory proteins and have anti-apoptotic and pro-apoptotic effects. Bcl-2-family proteins regulate mitochondrial membrane permeability, enabling cytochrome c release from mitochondria. Therefore, a high *Bax*/*Bcl-2* mRNA expression level ratio indicates apoptotic activity (Guicciardi and Gores, 2005).

**Figure 3:**
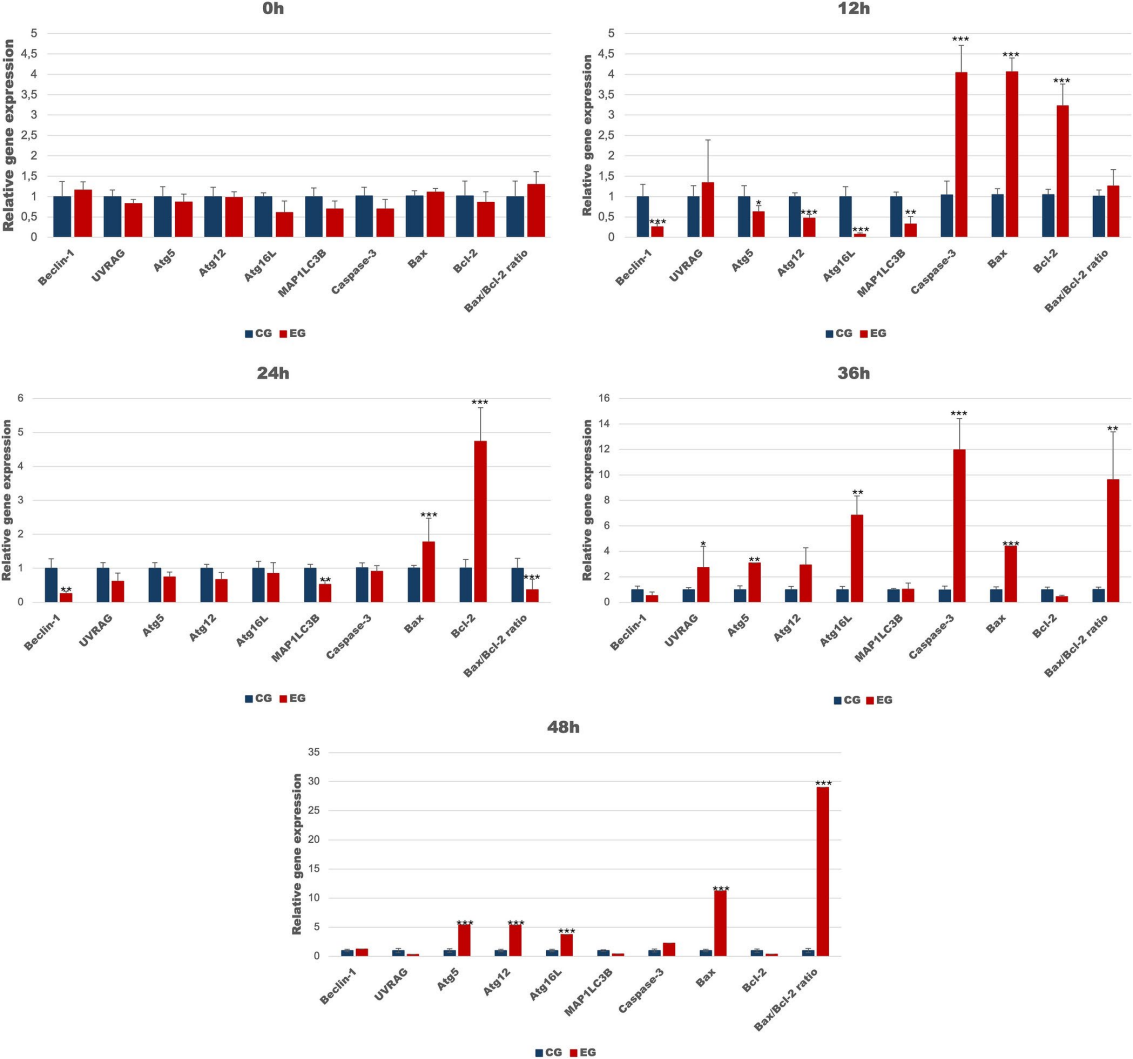
Expression levels of apoptotic and autophagic genes in blood samples in rabbits infected with *Lagovirus europaeus*/GI.2 at selected timepoints. All values are presented as the mean ± SE. ^*^p ≤ 0.05; ^**^p ≤ 0.03; ^***^p ≤ 0.01.

We assessed selected apoptotic markers to determine whether apoptotic gene expression changes in peripheral blood cells during *Lagovirus europaeus*/GI.2 infection (Table 2; Figure 3). We recorded at 12 h p.i. increased expression of *caspase-3* (*p* ≤ 0.01), as well as *Bax* (*p* ≤ 0.01) and *Bcl-2* (*p* ≤ 0.01), in infected rabbits compared to the control group. At 24 h p.i. there was an increase in the expression of anti-apoptotic *Bcl-2* (*p* ≤ 0.01), and reduced levels of *Bax*/*Bcl-2* mRNA expression ratio (*p* ≤ 0.01). Subsequently, we obtained increases in proapoptotic *Bax* at 36 h p.i. (*p* ≤ 0.05) and 48 h p.i. (*p* ≤ 0.01), and higher levels of *Bax*/*Bcl-2* mRNA expression ratio – 36 h p.i. (*p* ≤ 0.01) and 48 h p.i. (*p* ≤ 0.01), in addition to an increase in *caspase-3* gene expression only at 36 h p.i. (*p* ≤ 0.03).

**Table 2:** Results of apoptotic markers in blood samples determined by real-time PCR.

Gen	Parameter	Time after inoculation [h]									
		0		12		24		36		48	
	Group of animal	CG (*n* = 10)	EG (*n* = 10)	CG (*n* = 10)	EG (*n* = 10)	CG (*n* = 10)	EG (*n* = 8)	CG (*n* = 10)	EG (*n* = 4)	CG (*n* = 10)	EG (*n* = 1)
*Caspase-3*	Mean	1.02	0.70	1.04	4.04	1.02	0.91	0.98	11.98	0.99	2.27
	SD	0.68	0.72	1.08	2.10	0.46	0.54	0.92	4.24	0.79	–
	SE	0.21	0.23	0.34	0.67	0.14	0.17	0.29	2.45	0.25	–
	*p*-value	0.10		<0.01***		0.80		<0.01***		0.25	
*Bax*	Mean	1.02	1.11	1.05	4.06	1.01	1.78	1.00	4.41	0.99	11.26
	SD	0.39	0.27	0.45	1.07	0.27	2.46	0.70	1.75	0.72	–
	SE	0.12	0.09	0.14	0.34	0.08	0.70	0.22	0.01	0.23	–
	*p*- value	0.76		<0.01***		<0.01***		<0.01***		<0.01***	
*Bcl-2*	Mean	1.02	0.86	1.05	3.23	1.01	4.74	0.99	0.46	0.99	0.39
	SD	1.15	0.81	0.40	1.69	0.78	3.12	0.66	0.15	0.87	–
	SE	0.36	0.26	0.13	0.53	0.25	0.99	0.21	0.09	0.28	–
	*p*- value	0.57		<0.01***		<0.01***		0.09		0.36	
*Bax/Bcl-2 ratio*	Mean	1.00	1.30	1.01	1.26	1.00	0.37	1.01	9.63	1.00	29.01
	SD	1.19	0.98	0.46	1.27	0.93	0.99	0.59	6.51	1.16	–
	SE	0.38	0.31	0.15	0.40	0.30	0.31	0.19	3.76	0.37	–
	*p*- value	0.47		0.57		<0.01***		0.02**		<0.01***	

CG-control group; EG-experimental group; SD- standard deviation; SE- standard error; *p ≤ 0.05; **p ≤ 0.03; ***p ≤ 0.01.

### *Lagovirus europaeus*/GI.2 infection induces autophagic flux

The first stage of autophagy is the nucleation of the vesicles, and at this stage, the elements targeted for degradation are surrounded by an insulating membrane - phagophore. A key role during this phase plays a protein complex containing class III phosphatidylinositol 3-kinase (PI3K3 or Vps34), serine–threonine kinase (p150 or Vps15), and Beclin-1 (Atg6), which is directed to the site of autophagosome formation by Atg14 (Parzych and Klionsky, 2014). The activity of the PI3K3–Beclin-1–p150 complex can be regulated by a variety of proteins. The positive regulators of this complex include, among others UVRAG, which increases the activity of PI3K3 kinase and is involved in the maturation of the autophagosome (Bębnowska and Niedźwiedzka-Rystwej, 2022). The formation of the autophagosome is dependent on two ubiquitin-like protein systems. The first complex is formed by the conjugated Atg12 and Atg5 which then interact with Atg16L to form the Atg12–Atg5–Atg16L complex. The formation of this complex enables the elongation of the insulating membrane and thus the formation of an autophagosome (Parzych and Klionsky, 2014). The second complex necessary for autophagosome formation is LC3-II–PE. The light chain of microtubule-associated protein (LC3) is formed as a pro-LC3 precursor and transformed into the cytosolic form of LC3-I by proteolytic cleavage by Atg4. Through the activity of Atg7 and Atg3, the LC3-I form is linked with phosphatidylethanolamine (PE) and transforms into the mature LC3-II form, which is incorporated into the isolating membranes of autophagosomes. In the next step, LC3-II binds to the p62/SQSTM1 protein (Sequestosome 1), which recognizes ubiquitin located on the material to be degraded, creating a bridge between the autophagosome and the cargo to be removed (Parzych and Klionsky, 2014). The autophagosome is combined with the lysosome to form autolysosomes inside which the load is degraded (Parzych and Klionsky, 2014). The process of fusion of autophagosomes with lysosomes involves, among others, lysosomal associated membrane protein 1 (LAMP-1) and lysosomal associated membrane protein 2 (LAMP-2), which is why they serve as markers of late autophagic organelles (Klionsky et al., 2021).

**Figure 4:**
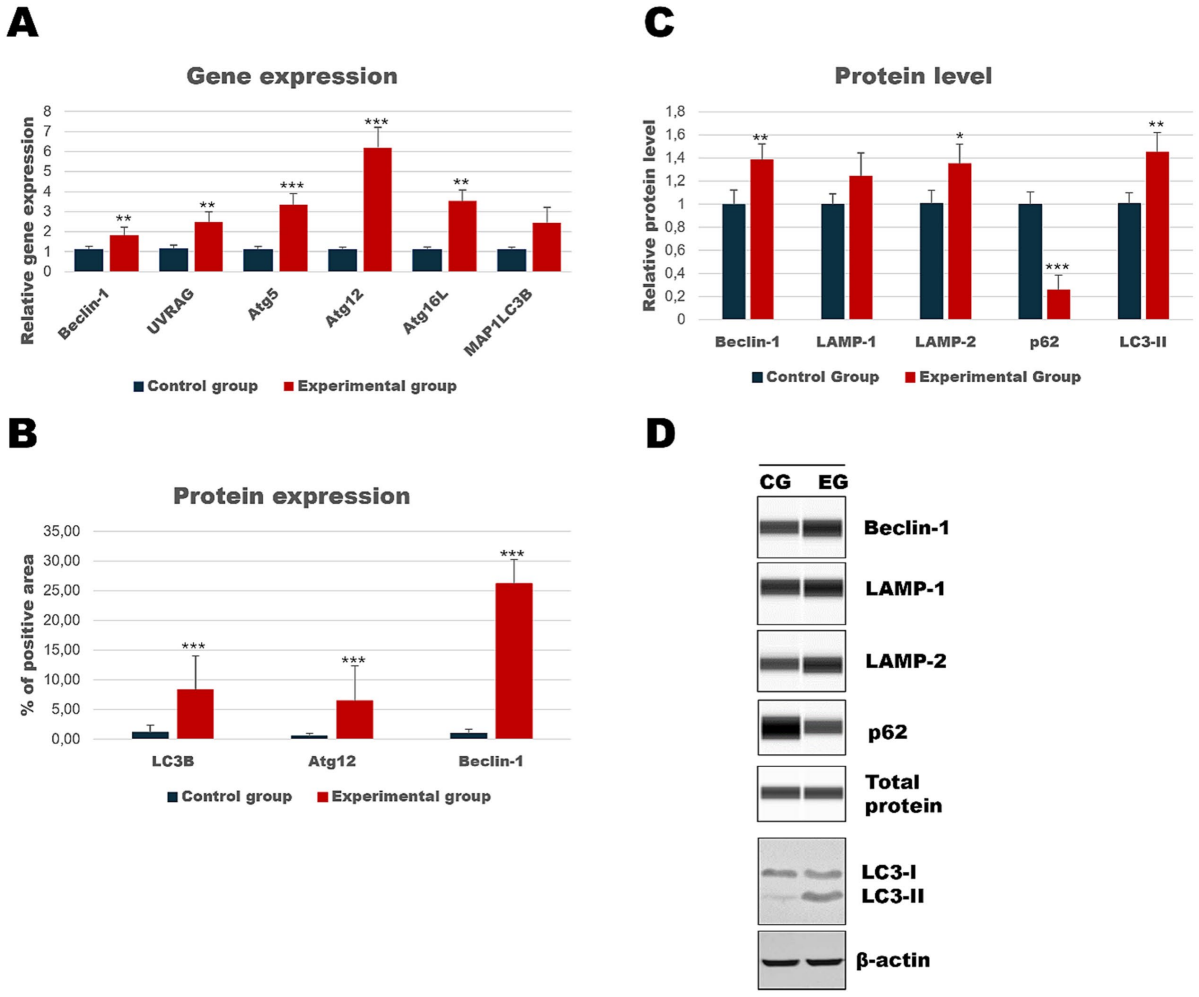
Results of autophagic markers analysis in the liver samples. The expression of selected autophagic genes is shown on panel **(A)**. Panel **(B)** shows the percentage of the immunopositive area obtained from computer analysis using ImageJ Fiji software (Johannes Schindelin, Albert Cardona, Mark Longair, Benjamin Schmid, and others, https://imagej.net/software/fiji/downloads, version 1.2) with support of the image analysis platform for immunohistochemically stained images SlideViewer. Relative levels of proteins measured by Western Blot method **(C)**. Panel **(D)** showed representative blots of autophagic proteins. The values are presented as the mean ± SE Panel **(A)** or SD Panel **(B,C)**. ^*^p ≤ 0.05; ^**^p ≤ 0.03; ^***^p ≤ 0.01. CG-control group; EG-experimental group.

**Figure 5:**
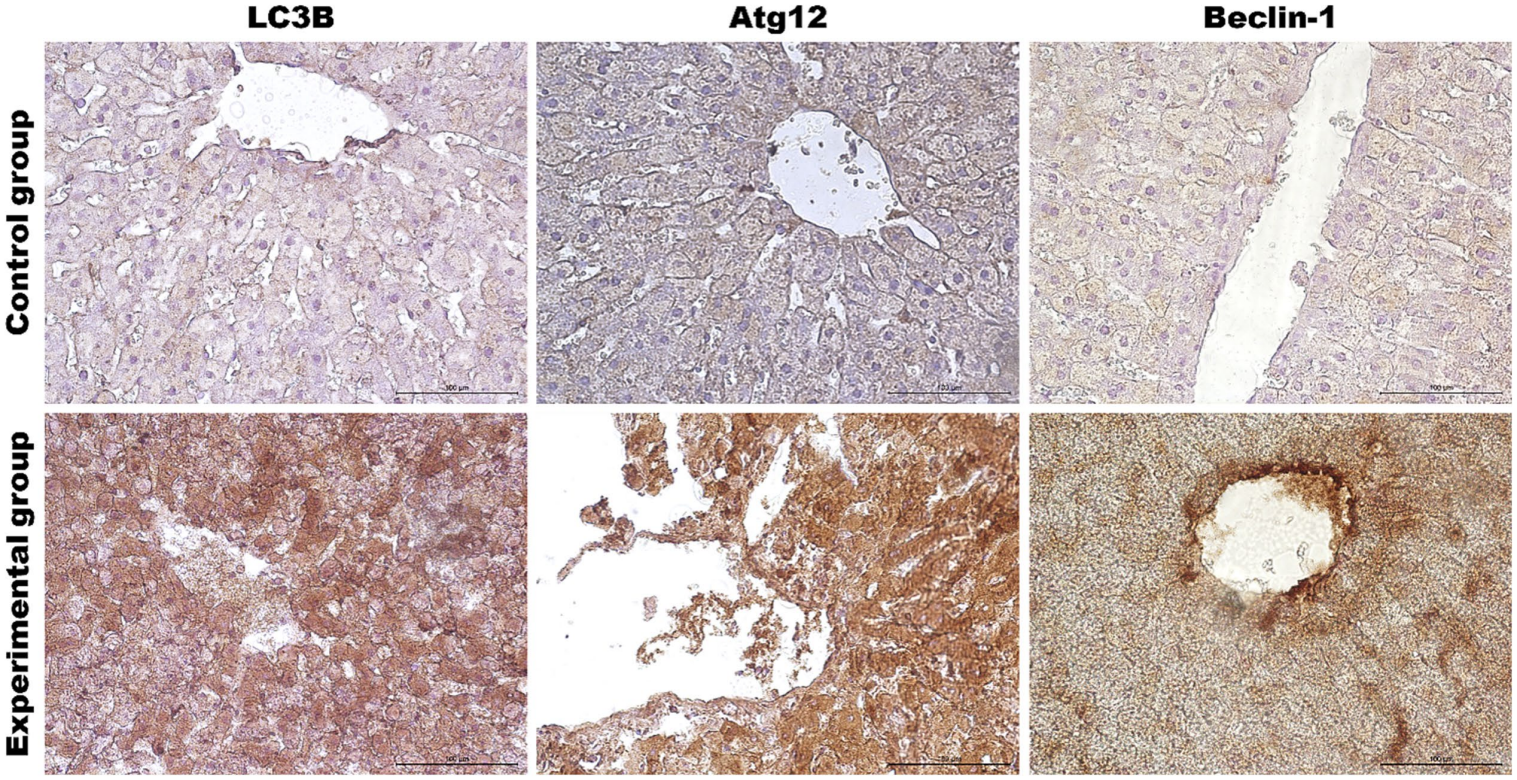
Representative pictures of selected autophagy markers (LC3B, Atg12, Beclin-1) in the liver from the control (upper panel) and *Lagovirus europaeus*/GI.2 (lower panel) infected rabbits. Immunohistochemical staining. Objective mag. X40, scale bar 100 μm. Leica DM5000B, Wetzlar, Germany.

As the liver is the main target of viral replication, we decided to analyze autophagic flux in this organ. Our results indicate that after the infection autophagic genes [*Beclin-1* (*p* ≤ 0.03), *UVRAG* (*p* ≤ 0.03), *Atg5* (*p* ≤ 0.01), *Atg12* (*p* ≤ 0.01) and *Atg16L* (*p* ≤ 0.03)] are upregulated in contrast to the control group (Table 3; Figure 4). We performed also immunohistochemical detection of three autophagic markers: LC3B, Beclin-1, and Atg12. Our results indicate that the expression of these three markers was higher in infected animals in contrast to the control group [LC3B (*p* ≤ 0.01), Beclin-1 (*p* ≤ 0.01), and Atg12 (*p* ≤ 0.01)] (Figure 4). Figure 5 shows the representative pictures of a comparison between the experimental and control groups. We also assessed the levels of key proteins involved in autophagy. Our results indicate that in the liver of infected rabbits compared to the control group there is an increased level of Beclin-1 (*p* ≤ 0.03), LAMP-2 (*p* ≤ 0.05), LC3-II (*p* ≤ 0.03), but also a decreased level of p62 protein (*p* ≤ 0.01), indicating an autophagic flux in this organ (Figure 4).

**Table 3:** Results of autophagic markers in selected organs determined by real-time PCR.

Gen	Parameter	Organ					
		Liver		Spleen		Kidney	
	Group of animals	CG (*n* = 10)	EG (*n* = 10)	CG (*n* = 10)	EG (*n* = 10)	CG (*n* = 10)	EG (*n* = 10)
*Beclin-1*	Mean	1.12	1.82	1.09	4.39	1.18	1.53
	SD	0.52	0.41	0.49	3.06	0.60	0.36
	SE	0.16	1.30	0.15	0.97	0.18	0.11
	*p*- value	0.13		<0.01***		0.18	
*UVRAG*	Mean	1.13	2.48	1.03	2.04	1.04	2.01
	SD	0.60	0.51	0.30	1.37	0.31	0.86
	SE	0.19	1.63	0.09	0.43	0.09	0.27
	*p*- value	0.02**		0.03*		0.02**	
*Atg5*	Mean	1.09	3.35	1.25	4.94	1.04	3.55
	SD	0.48	1.76	1.19	3.14	0.34	1.75
	SE	0.15	0.56	0.38	0.99	0.10	0.55
	*p*- value	<0.01***		<0.01***		<0.01***	
*Atg12*	Mean	1.08	6.20	1.41	18.47	1.10	3.47
	SD	0.42	3.24	0.69	15.16	0.15	2.36
	SE	0.13	1.02	0.21	4.79	0.49	0.75
	*p*- value	<0.01***		<0.01***		<0.01***	
*Atg16L*	Mean	1.10	3.53	1.10	4.74	1.08	1.65
	SD	0.45	1.78	0.50	2.33	0.49	0.79
	SE	0.14	0.56	0.15	0.74	0.15	0.25
	*p*- value	<0.01***		<0.01***		0.04*	
*MAP1LC3B*	Mean	1.09	2.44	1.04	2.20	1.04	2.73
	SD	0.40	0.78	0.33	1.20	0.32	1.73
	SE	0.13	2.49	0.10	0.38	0.10	0.55
	*p*- value	0.11		<0.01***		0.08	

CG-control group; EG-experimental group; SD- standard deviation; SE- standard error; *p ≤ 0.05 **p ≤ 0.03; ***p ≤ 0.01.

**Figure 6:**
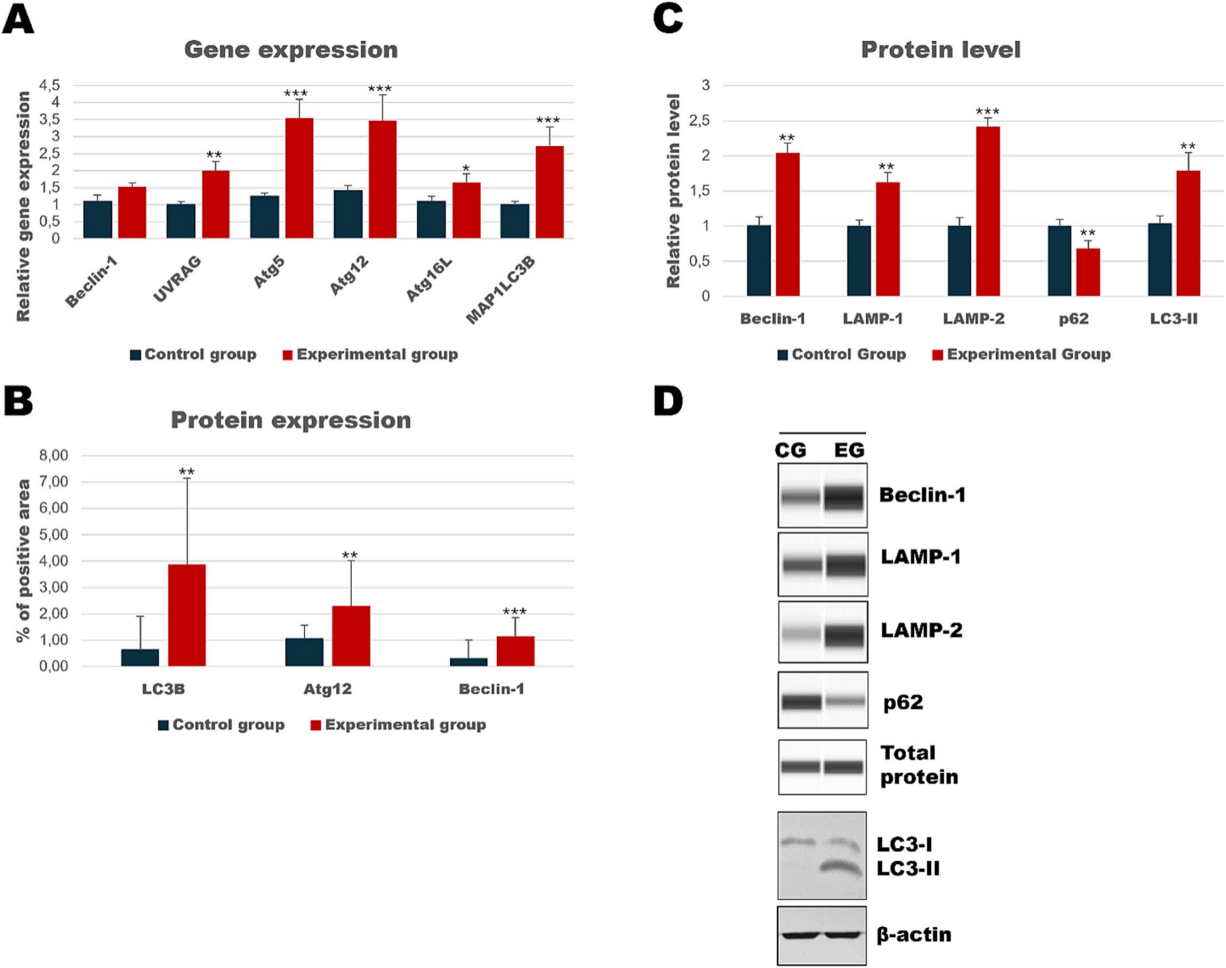
Results of autophagic markers analysis in the kidney samples. The expression of selected autophagic genes is shown on panel **(A)**. Panel **(B)** shows the percentage of the immunopositive area obtained from computer analysis using ImageJ Fiji software (Johannes Schindelin, Albert Cardona, Mark Longair, Benjamin Schmid, and others, https://imagej.net/software/fiji/downloads, version 1.2) with support of the image analysis platform for immunohistochemically stained images SlideViewer. Relative levels of proteins measured by Western Blot method **(C)**. Panel **(D)** showed representative blots of autophagic proteins. The values are presented as the mean ± SE Panel **(A)** or SD Panel **(B,C)**. ^*^p ≤ 0.05; ^**^p ≤ 0.03; ^***^p ≤ 0.01. CG-control group; EG-experimental group.

**Figure 7:**
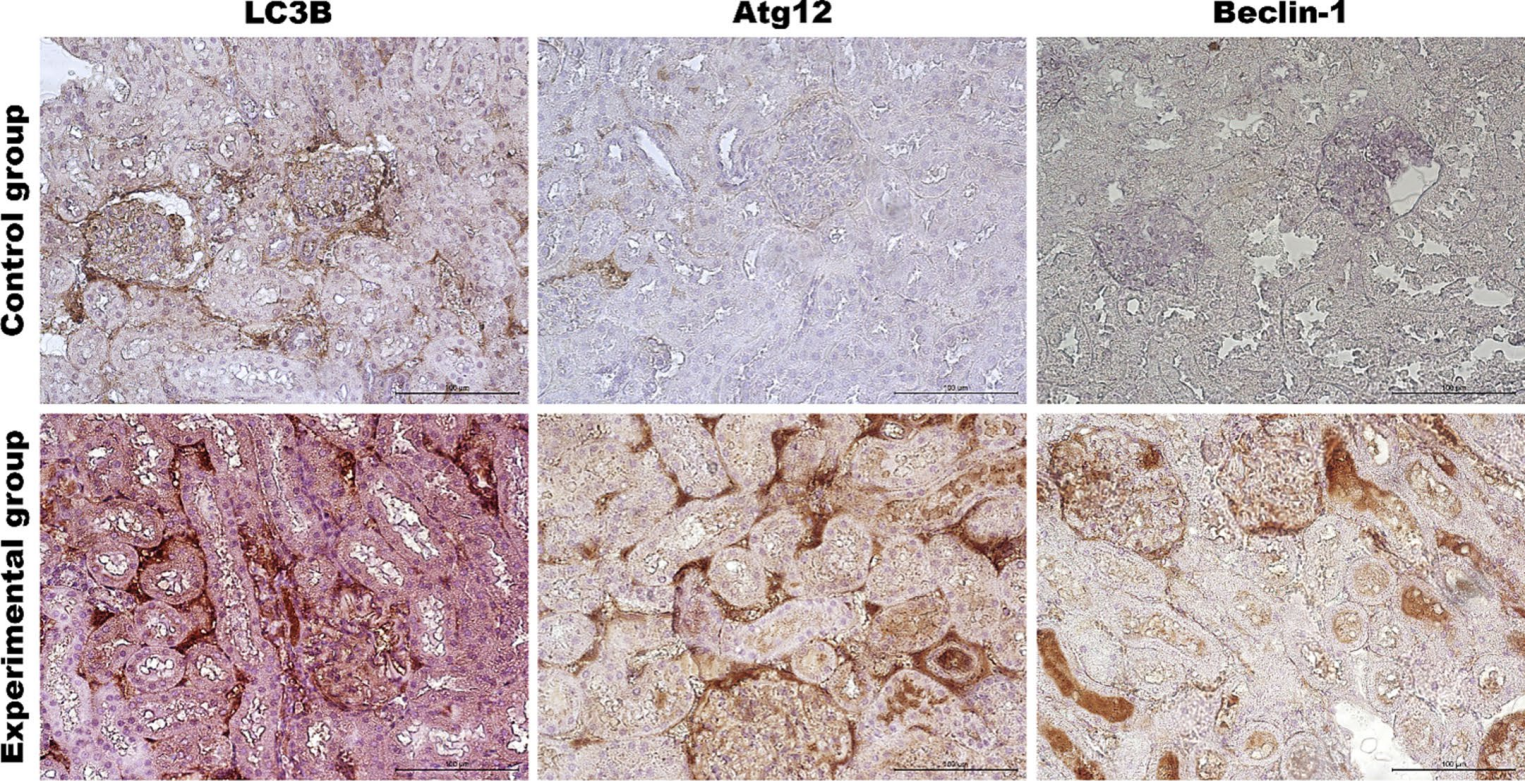
Representative pictures of selected autophagy markers (LC3B, Atg12, Beclin-1) in the renal cortex from the control (upper panel) and *Lagovirus europaeus*/GI.2. (lower panel) infected rabbits. Immunohistochemical staining. Objective mag. X40, scale bar 100 μm. Leica DM5000B, Wetzlar, Germany.

**Figure 8:**
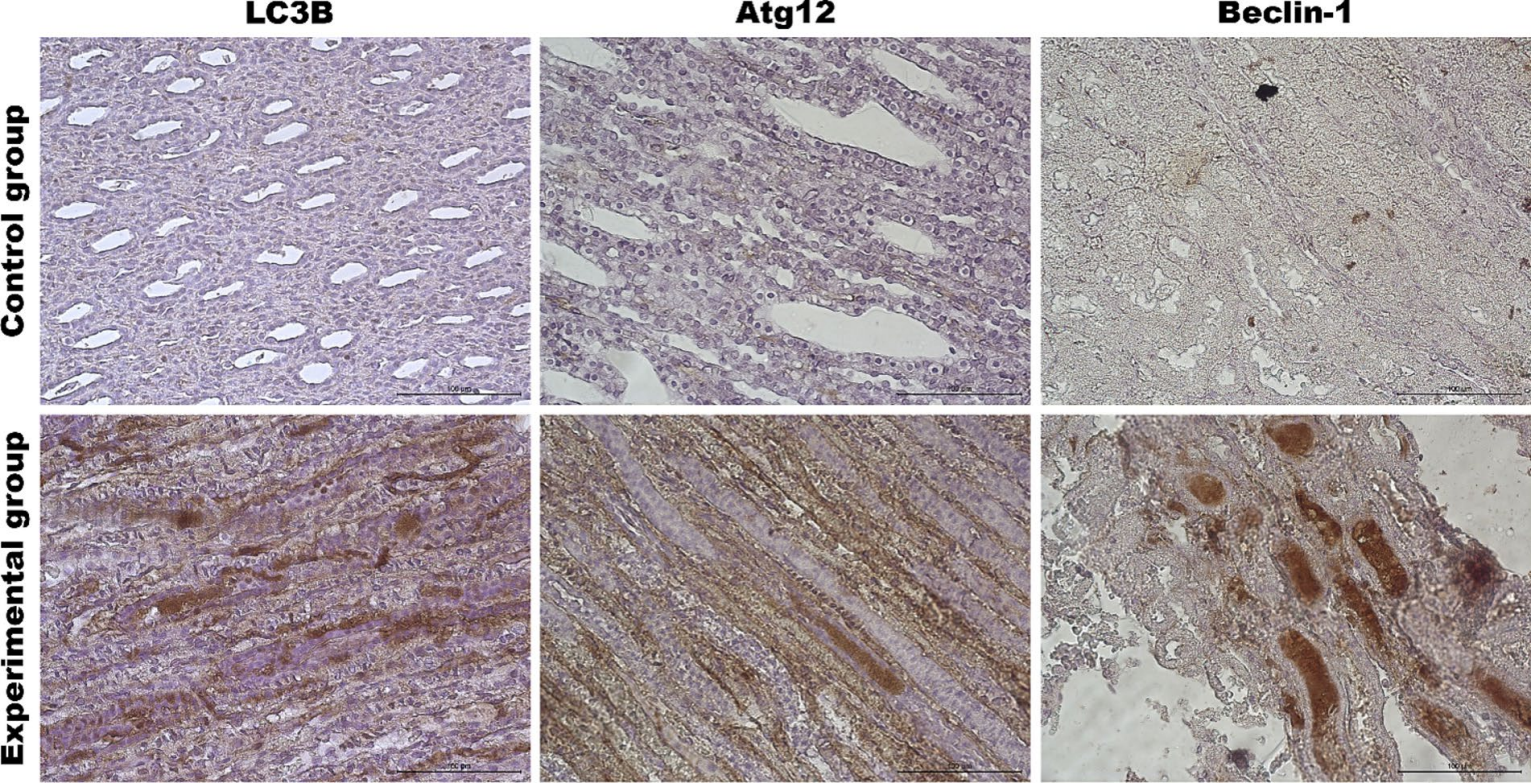
Representative pictures of selected autophagy markers (LC3B, Atg12, Beclin-1) in the renal medulla from the control (upper panel) and *Lagovirus europaeus*/GI.2. (lower panel) infected rabbits. Immunohistochemical staining. Objective mag. X40, scale bar 100 μm. Leica DM5000B, Wetzlar, Germany.

Our investigation also included two additional organs because we have lately shown a strong apoptotic response in kidneys and spleen tissue during *Lagovirus europaeus*/GI.2 infection (Bębnowska et al., 2023). We looked at whether the autophagic response is equally noticeable in these organs. Results showed that in the kidney tissue of infected rabbits, some autophagic genes are upregulated [*UVRAG* (*p* ≤ 0.03), *Atg5* (*p* ≤ 0.01), *Atg12* (*p* ≤ 0.01)*, Atg16L* (*p* ≤ 0.05) and *MAP1LC3B* (*p* ≤ 0.01)] compared to the control group (Table 3, Figure 6). Immunohistochemical assay indicated high expression of LC3B (*p* ≤ 0.03), Atg12 (*p* ≤ 0.03), and Beclin-1 (*p* ≤ 0.01) proteins and changes were detected in the renal cortex and renal medulla (Figures 6–8). Additional analysis of relative protein levels indicated elevated values of Beclin-1 (*p* ≤ 0.03), LAMP-1 (*p* ≤ 0.03), LAMP-2 (*p* ≤ 0.01), and LC3-II (*p* ≤ 0.03), but also decreased level of p62 (*p* ≤ 0.03) compared to the control group (Figure 6).

**Figure 9:**
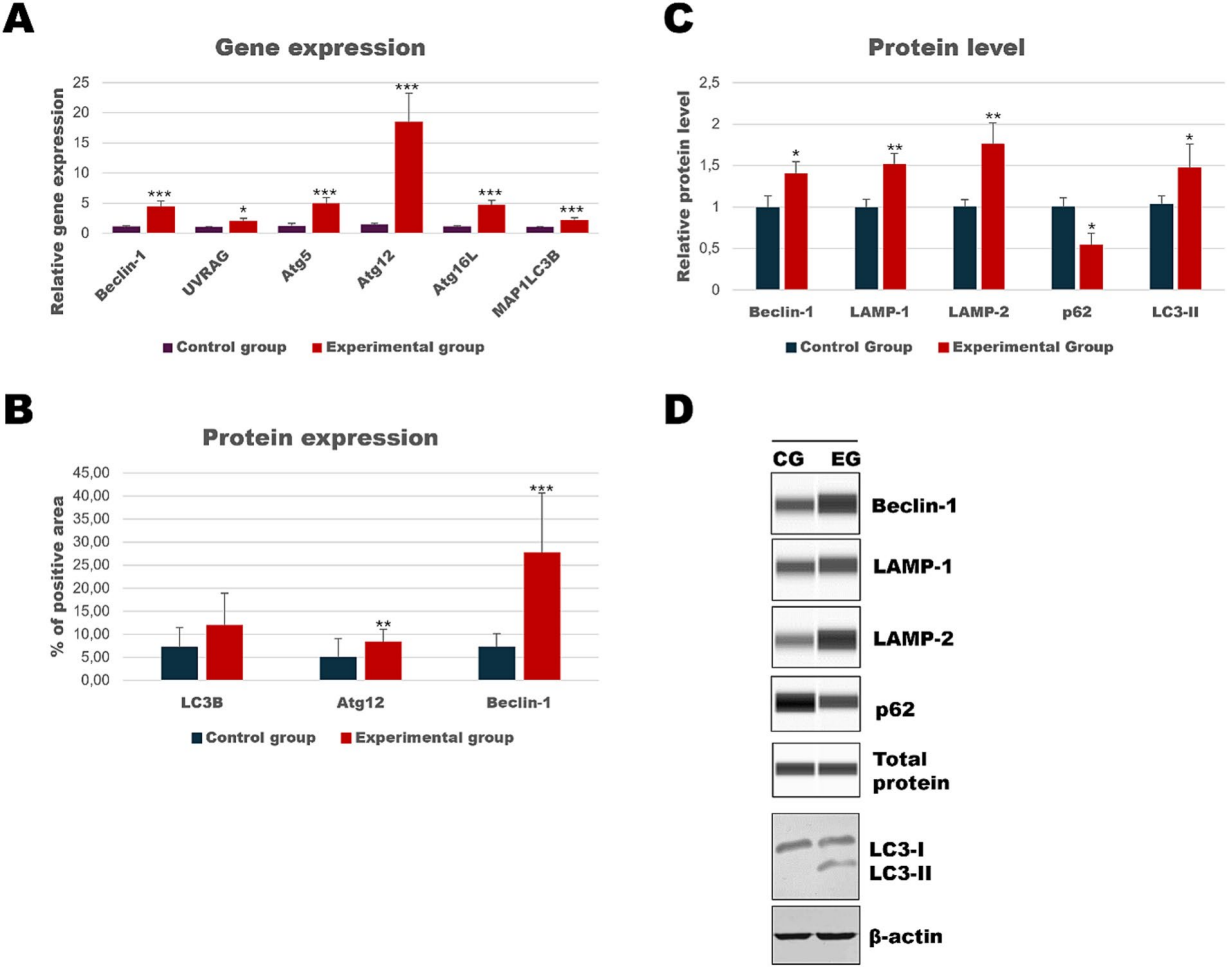
Results of autophagic markers analysis in the spleen samples. The expression of selected autophagic genes is shown on panel **(A)**. Panel **(B)** shows the percentage of the immunopositive area obtained from computer analysis using ImageJ Fiji software (Johannes Schindelin, Albert Cardona, Mark Longair, Benjamin Schmid, and others, https://imagej.net/software/fiji/downloads, version 1.2) with support of the image analysis platform for immunohistochemically stained images SlideViewer. Relative levels of proteins measured by Western Blot method **(C)**. Panel **(D)** showed representative blots of autophagic proteins. The values are presented as the mean ± SE Panel **(A)** or SD Panel **(B,C)**. ^*^p ≤ 0.05; ^**^p ≤ 0.03; ^***^p ≤ 0.01. CG-control group; EG-experimental group.

**Figure 10:**
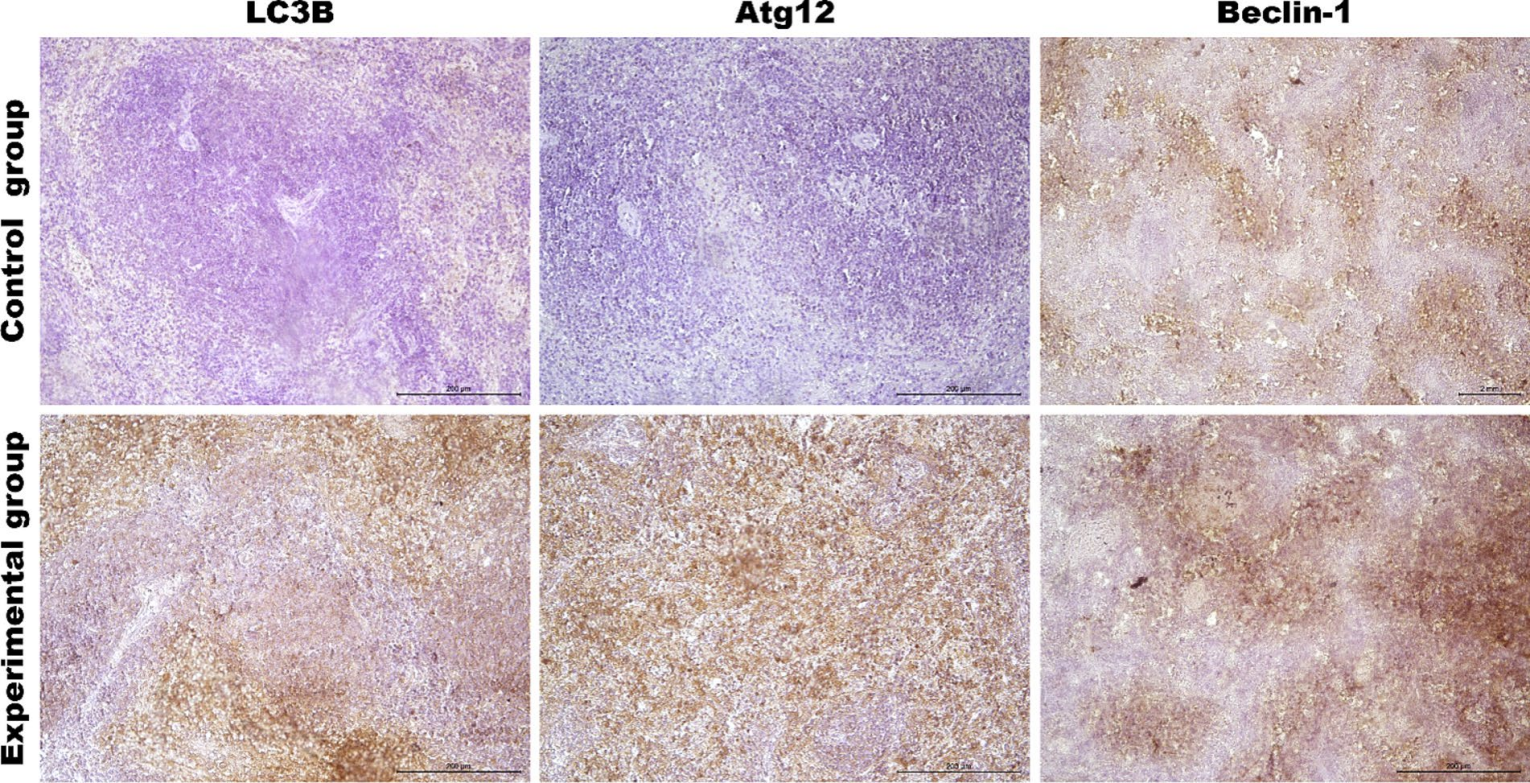
Representative pictures of selected autophagy markers (LC3B, Atg12, Beclin-1) in the spleen from the control (upper panel) and *Lagovirus europaeus*/GI.2. (lower panel) infected rabbits. Immunohistochemical staining. Objective mag. X40, scale bar 100 μm. Leica DM5000B, Wetzlar, Germany.

Analysis of spleen tissue showed that autophagic genes [*Beclin-1* (*p* ≤ 0.01), *UVRAG* (*p* ≤ 0.05), *Atg5* (*p* ≤ 0.01), *Atg12* (*p* ≤ 0.01), *Atg16L* (*p* ≤ 0.01) and *MAP1LC3B* (*p* ≤ 0.01)] were upregulated in the experimental group, and also a high expression of Beclin-1 (*p* ≤ 0.01) and Atg12 (*p* ≤ 0.03) proteins (Table 3; Figure 9) was detected. Figure 10 shows representative pictures of the result from the immunohistochemical assay. Results of relative protein levels analysis of autophagic markers determined by the Western Blot method showed in the spleen tissue higher values of Beclin-1 (*p* ≤ 0.05), LAMP-1 (*p* ≤ 0.03), LAMP-2 (*p* ≤ 0.03), and LC3-II (*p* ≤ 0.05), but also a lower level of p62 (*p* ≤ 0.05) compared to the control group.

To determine whether autophagic genes are upregulated in peripheral blood cells during *Lagovirus europaeus*/GI.2 infection, we assessed the expression of several key markers (Table 4; Figure 2). Our results indicate that in the experimental group, compared to the healthy control, there is a decrease in the expression of autophagy-related genes during the initial stage of infection, which increases over time. We registered that the mRNA expression of *Beclin-1* (*p* ≤ 0.01), *MAP1LC3B* (*p* ≤ 0.03), *Atg5* (*p* ≤ 0.05), *Atg12* (*p* ≤ 0.01) and *Atg16L* (*p* ≤ 0.03) was decreased in the experimental group compared to the control group at 12 h p.i. At 24 h p.i. decrease in autophagy gene expression was ongoing and significant differences were registered in *Beclin-1* (*p* ≤ 0.03) and *MAP1LC3B* (*p* ≤ 0.03). From 36 h p.i. there was an increase in *UVRAG* (*p* ≤ 0.05), *Atg5* (*p* ≤ 0.03), and *Atg16L* (*p* ≤ 0.03) mRNA expression. At 48 h p.i., higher levels of *Atg5*(*p* ≤ 0.01), *Atg12* (*p* ≤ 0.01), and *Atg16L* (*p* ≤ 0.01) were obtained in the experimental group compared to the control.

**Table 4:** Expression levels of autophagic genes in blood samples determined by real-time PCR.

Gen	Parameter	Time after inoculation									
		0 h		12 h		24 h		36 h		48 h	
	Group	CG (*n* = 10)	EG (*n* = 10)	CG (*n* = 10)	EG (*n* = 10)	CG (*n* = 10)	EG (*n* = 8)	CG (*n* = 10)	EG (*n* = 4)	CG (*n* = 10)	EG (*n* = 1)
*Beclin-1*	Mean	1.00	1.16	1.00	0.26	1.00	0.26	1.00	0.54	1.00	1.27
	SD	1.16	0.64	0.96	0.26	0.89	0.22	0.87	0.47	0.72	–
	SE	0.37	0.20	0.30	0.08	0.28	0.07	0.28	0.27	0.23	–
	*p*- value	0.63		<0.01***		0.02**		0.21		0.93	
*UVRAG*	Mean	1.00	0.83	1.00	1.34	1.00	0.62	1.00	2.74	1.00	0.35
	SD	0.50	0.31	0.85	3.32	0.55	0.77	0.51	2.88	1.08	–
	SE	0.16	0.10	0.27	1.05	0.17	0.24	0.16	1.66	0.34	–
	*p*- value	0.26		0.57		0.33		0.03*		0.41	
*Atg5*	Mean	1.00	0.87	1.00	0.63	1.00	0.75	1.00	3.09	1.00	5.42
	SD	0.74	0.60	0.84	0.47	0.53	0.44	0.93	0.17	0.93	–
	SE	0.24	0.19	0.27	0.15	0.17	0.14	0.29	0.01	0.29	–
	*p*- value	0.27		0.04*		0.11		0.02**		<0.01***	
*Atg12*	Mean	1.00	0.98	1,00	0.47	1.00	0.67	1.00	2.94	1.00	5.39
	SD	0.71	0.45	0,28	0.29	0.39	0.66	0.83	2.32	0.63	–
	SE	0.23	0.14	0,09	0.09	0.12	0.21	0.26	1.34	0.20	–
	*p*- value	0.30		<0.01***		0.61		0.07		<0.01***	
*Atg16L*	Mean	1.00	0.61	1.00	0.08	1.00	0.85	1.00	6.84	1.00	3.75
	SD	0.29	0.88	0.76	0.09	0.68	1.01	0.78	2.63	0.66	–
	SE	0.09	0.28	0.24	0.03	0.21	0.32	0.25	1.52	0.21	–
	*p*- value	0.95		<0.01***		0.73		0.02**		<0.01***	
*MAP1LC3B*	Mean	1.00	0.70	1.00	0.33	1.00	0.53	1.00	1.04	1.00	0.43
	SD	0.67	0.61	0.36	0.58	0.39	0.34	0.31	0.83	0.46	–
	SE	0.21	0.19	0.11	0.18	0.12	0.11	0.10	0.48	0.15	–
	*p*- value	0.22		0.02**		0.02**		0.57		0.20	

CG-control group; EG-experimental group; SD- standard deviation; SE- standard error; *p ≤ 0.05; **p ≤ 0.03; ***p ≤ 0.01.

## Discussion

Autophagy and apoptosis are two immunological processes that play key roles in viral infection by being part of host antiviral immunity, but may also be linked to disease pathogenesis. Autophagy is activated in the cell to relieve the stress stimulus and restore homeostatic stability. However, if the stress does not subside, the cells may activate apoptotic machinery that enables their elimination with limited induction of local inflammation (Booth et al., 2014). Together, these two processes determine the future fate of the cell and many points at which autophagy and apoptosis can interact have been confirmed and have been described in detail in many reviews (Mariño et al., 2014; Booth et al., 2014; Nikoletopoulou et al., 2013).

Apoptosis has been described as one of the primary pathomechanisms of the disease in Rabbit Haemorrhagic Disease. In our previous studies, we have shown that rabbits infected with the GI.2 strain have increased expression of *caspase-3*, *Bax*, *Bcl-2* and the *Bax*/*Bcl-2* mRNA gene expression ratio in the spleen, kidney, lung and heart of infected animals. Our results also showed increased levels of cleaved caspase-3, caspase-6 and PARP, as well as significant caspase-3 activity (Bębnowska et al., 2023). The liver is the central organ of viral replication, so it is a major subject of research toward a better understanding of this disease. In a previous study, we demonstrated the presence of morphological signs of apoptosis in rabbit hepatocytes, and our results suggested that caspase-3 and Bcl-2 family proteins may play an important role in the apoptotic cellular debris induced by *Lagovirus europaeus* /GI.2 infection (Bębnowska et al., 2024). In this study, we confirmed an increase in the levels of cleaved caspase-3, caspase-6 and PARP and increased caspase-3 activity in rabbits from the experimental group compared to the control.

In the present study, we analyzed the expression of genes related to apoptotic cell death in blood samples. We recorded that at 12 and 36 h p.i. there was increased expression of *caspase-3* in infected rabbits compared to the control group. In addition, infection was accompanied by significant changes in the expression of proapoptotic *Bax* and anti-apoptotic *Bcl-2*. We found increased expression of *Bax* at 12, 36 and 48 h p.i. and increased expression of *Bcl-2* at 12 and 24 h p.i., which was decreased at 36 and 48 h p.i. In addition, the determined *Bax*/*Bcl-2* ratio values were higher at 36 and 48 h p.i. and significantly lower at 24 h p.i. These results may suggest that apoptosis occurred in infected rabbits from 12 h p.i. and was attenuated at 24 h p.i., which is also confirmed by reduced *caspase-3* expression at this time point. Previous studies (Niedźwiedzka-Rystwej and Deptuła, 2023; Niedźwiedzka-Rystwej and Deptuła, 2012; Niedźwiedzka-Rystwej et al., 2013) in *Lagovirus europaeus*/GI.1 infection showed that apoptosis was detected in peripheral blood granulocytes and lymphocytes from 8 or 12 h p.i. in haemagglutinogenic strains (24 V, 1447 V/96, 01–04, 237/04, V-412, 05–01) and from 4 h p.i. in non-haemagglutinogenic strains (Rainham, Frankfurt and Asturias). This indicates that the infection involved the incorporation of apoptotic cell death from the beginning of the infection, which coincides with the results obtained in the present study. Also importantly, it was shown (Niedźwiedzka-Rystwej et al., 2013) that the number of apoptotic granulocytes (01–04 and 1,447 V/96 strain) and lymphocytes (72 V/2003 and 1447/96 strain) decreased at 24 h p.i. for some strains and then increased at 36 h p.i. This observation is in agreement with our speculation based on results obtained in this study, where we recorded low *caspase-3* expression at 24 h p.i. and a strong up-regulation of anti-apoptotic *Bcl-2* (accompanied by a reduced *Bax*/*Bcl-2* mRNA expression ratio).

In this study, we also evaluated the activation of the autophagic pathway in *Lagovirus europaeus*/GI.2 infection. For our analysis, we selected a few markers that are involved in the autophagic pathway at different stages. Our analysis focused on several organs, including the liver, which is the central site of viral replication, but also the kidney and spleen. As a result, we showed that there is an up-regulation of key autophagy genes in infected rabbits: *Beclin-1* (liver, spleen), *UVRAG* (liver, kidney, spleen), *Atg5* (liver, kidney, spleen), *Atg12* (liver, kidney, spleen), *Atg16L* (liver, kidney, spleen), *MAP1LC3B* (kidney, spleen). In this study, we also analyzed autophagic protein markers. Immunohistochemical analysis showed that the expression of several proteins encoded by these genes was significantly higher in the tissues of infected rabbits: Beclin-1 (liver, kidney, spleen), Atg12 (liver, kidney, spleen) and LC3B (liver, kidney). Our results also showed increased relative levels of selected proteins in infected rabbits compared to controls: Beclin-1 (liver, kidney, spleen), LAMP-1 (kidney, spleen), LAMP-2 (liver, kidney, spleen), and LC3-II (liver, kidney, spleen). In addition, we also assessed the amount of p62 protein, which is associated with the process of dosing the autophagosome with cargo destined for degradation. The p62 protein loaded with ubiquitinated proteins binds to LC3-II and, after forming the autophagosome and then the autolysosome, is degraded in the final phase of autophagy (Parzych and Klionsky, 2014; Klionsky et al., 2021). For this reason, it is a good indicator of total autophagic flux. In this study, we showed that p62 protein levels were lower in infected rabbits compared to controls in all organs analyzed, indicating total autophagy. Given that apoptosis of peripheral blood granulocytes and lymphocytes is an important component of the pathogenesis of RHD, we also decided to preliminarily assess whether there is also altered activity of autophagy markers in infected rabbits. As a result, we demonstrated at 12 h p.i. a decrease in the expression of autophagy-related genes (*Beclin-1*, *Atg5*, *Atg12*, *Atg16L,* and *MAP1LC3B*). Subsequently, at 24 h p.i., we recorded decreased expression of *Beclin-1* and *MAP1LC3B*. In contrast, up-regulation of gene expression was observed at 36 h p.i. (*UVRAG*, *Atg5*, *Atg16L*) and at 48 h p.i. (*Atg5*, *Atg12*, *Atg16L*).

Autophagy was analyzed in the liver of rabbits infected with *Lagovirus europaeus*/GI.1 by Vallejo et al. (2014). The authors showed that an increased number and content of autophagic vesicles was detectable in cells from 12 h p.i. The study also assessed the expression of key genes involved in autophagic flux. It was recorded that *Beclin-1* was up-regulated at 18 and 24 h p.i., and *UVRAG* was higher at 24 h p.i., after which its levels began to decrease. *Atg5* and *Atg12* expression increases from 12 h p.i. and 18 h p.i. *Atg16L*. Furthermore, expression of the *p62/SQSTM* gene was increased from 12 to 24 h p.i (Vallejo et al., 2014). Although up-regulation of autophagy genes is common in viral infections, there are reports in which a decrease in the expression of molecular markers of autophagy accompanied virus infection. Tomić et al. (2021) showed that in COVID-19 patients, transcript levels of autophagy-promoting factors including *ATG5*, *UVRAG,* and *LC3* were significantly decreased in PBMCs compared to controls, and this phenomenon was more pronounced in patients with severe disease. Furthermore, *Beclin-1* and *p62/SQSTM* expression was not significantly altered, but positively correlated with the other genes (Tomić et al., 2021).

The activity of mTOR kinase largely controls autophagic flux. Under normal conditions, mTOR kinase negatively regulates the expression of autophagy genes and, due to stress stimuli, activates autophagic flux. The induction of mTOR signaling has been reported to occur in viral infections (He, 2006). Vallejo et al. (2014) showed that in *Lagovirus europaeus*/GI.1 infection there is increased expression of phospho-mTOR in the liver at 12, 18, and 24 h p.i., which unexpectedly correlated with the simultaneous development of autophagy. These results indicated that mTOR was not a negative regulator of autophagic flux. Given that our study achieved down-regulation of autophagy genes (*Beclin-1*, *Atg5*, *Atg12*, *Atg16L,* and *MAP1LC3B*) in blood cells during the initial phase of infection, further work will be required to determine the involvement of mTOR signaling in this phenomenon.

In general, the autophagy mechanism is activated in the cell earlier than apoptosis, which is confirmed in various viral infections (Sun et al., 2022; Zhou et al., 2021). Zhou et al. (2021) reported autophagy activation in carp populosum epithelioma cells infected with *Siniperca chuatsi* Rhabdovirus, and this process delayed the onset of apoptotic cell death. Autophagy may also contribute to the induction of apoptosis. In a study by Yeganeh et al. (2018), inhibition of autophagy with 3-methyladenine (3-MA) resulted in blocking intrinsic apoptotic signaling in an *in vitro* model of influenza A virus infection. Also, Ma et al. (2011) showed that the knockout of the *Beclin-1* gene and the treatment with the autophagy inhibitor 3-MA resulted in a significant reduction in avian influenza H5N1 virus-induced cell death in primary mouse fibroblasts (MEFs). As mentioned earlier, Vallejo et al. (2014) showed that in *Lagovirus europaeus*/GI.1 infection, autophagy was present during the initial phase of infection, while when autophagy decreased at 36–48 h p.i., the apoptotic response was enhanced. This indicates that autophagy protects against the damaging effects of virus-induced apoptosis (Vallejo et al., 2014).

We recorded a strong expression of apoptotic genes in the peripheral blood during the initial phase of infection, and from 36 h p.i. up-regulation of autophagy markers occurred. This may suggest that autophagy did not play a protective role against apoptosis in blood cells in infected rabbits. Sun et al. (2022) reported that in the cell line from the porcine kidney (PK-15) after infection with pseudorabies virus (PRV), autophagy activity was observed at 2 h p.i., while apoptosis was demonstrated at 12 h p.i., which was the key switch point between these processes. Moreover, PRV-induced apoptosis was enhanced during autophagy inhibition, while autophagy enhancement inhibited apoptosis. We speculate, therefore, that such a rapid apoptotic response in blood cells in rabbits infected with *Lagovirus europaeus*/GI.2 may have some relation to the possible inhibition of autophagy during the initial phase of infection, and that the attenuation of the apoptotic response at 24 h p.i. may be associated with the up-regulation of autophagy genes. In the future, it is also important to look at other immune pathological phenomena such as cytokine storm, which can contribute to the death of an animal, and has been well documented in some viral infections, including viral haemorrhagic fevers. This is also important because a large activation of pro- and anti-inflammatory cytokines has been documented in *Lagovirus europaeus*/GI.1 infection (Müller et al., 2021). However, a further experiment has to be performed in the future to investigate this issue, because it seems to be an interesting research direction.

However, our study has some limitations. Given the strong dynamics of changes in molecular markers, it would be appropriate to extend future experiments to include more time points at which material is collected, especially in the early phase of infection (especially between 0 and 24 h of the experiment). We note the strong need to more accurately determine changes in molecular markers on the first day of infection, as it is important to keep in mind animals that died earlier without symptoms. This is also important since Rabbit Haemorrhagic Disease infection can occur in various clinical forms of the disease and with a high mortality rate in the early hours (as shown in this study). Hall et al. (2021) reported that GI.2 strains are highly virulent in previously unvaccinated laboratory rabbits regardless of the infectious dose, so it would be worth considering in the future to apply a route of virus administration other than intramuscular, e.g., imitating a natural infection. The experimental dose of virus used to provoke infection should also be questioned. A high dose of virus can lead to systemic damage that leads to the death of the animal, regardless of cellular mechanisms such as those described in this work. Therefore, it is necessary to conduct further studies in the future to verify these issues. However, this is debatable as our previous experiments analyzing immune parameters in rabbits infected with *Lagovirus europaeus* include a common RHD challenge regimen, making the results more comparable.

## Data Availability

The datasets presented in this study can be found in online repositories. The names of the repository/repositories and accession number(s) can be found in the article/supplementary material.

## Footnotes

^1^Johannes Schindelin, Albert Cardona, Mark Longair, Benjamin Schmid, and others, https://imagej.net/software/fiji/downloads, version 1.2.
